# SEGMA: An Automatic SEGMentation Approach for Human Brain MRI Using Sliding Window and Random Forests

**DOI:** 10.3389/fninf.2017.00002

**Published:** 2017-01-20

**Authors:** Ahmed Serag, Alastair G. Wilkinson, Emma J. Telford, Rozalia Pataky, Sarah A. Sparrow, Devasuda Anblagan, Gillian Macnaught, Scott I. Semple, James P. Boardman

**Affiliations:** ^1^MRC Centre for Reproductive Health, University of EdinburghEdinburgh, UK; ^2^Department of Radiology, Royal Hospital for Sick ChildrenEdinburgh, UK; ^3^Centre for Clinical Brain Sciences, University of EdinburghEdinburgh, UK; ^4^Clinical Research Imaging Centre, University of EdinburghEdinburgh, UK; ^5^Centre for Cardiovascular Science, University of EdinburghEdinburgh, UK

**Keywords:** brain, MRI, large-scale, life-course, sliding window, random forests, classification, tissue segmentation

## Abstract

Quantitative volumes from brain magnetic resonance imaging (MRI) acquired across the life course may be useful for investigating long term effects of risk and resilience factors for brain development and healthy aging, and for understanding early life determinants of adult brain structure. Therefore, there is an increasing need for automated segmentation tools that can be applied to images acquired at different life stages. We developed an automatic segmentation method for human brain MRI, where a sliding window approach and a multi-class random forest classifier were applied to high-dimensional feature vectors for accurate segmentation. The method performed well on brain MRI data acquired from 179 individuals, analyzed in three age groups: newborns (38–42 weeks gestational age), children and adolescents (4–17 years) and adults (35–71 years). As the method can learn from partially labeled datasets, it can be used to segment large-scale datasets efficiently. It could also be applied to different populations and imaging modalities across the life course.

## Introduction

During early life, the brain undergoes significant morphological and functional changes, the integrity of which determines long-term neurological, cognitive and psychiatric functions (Tamnes et al., 2013). For instance, a wide range of problems including autism spectrum disorder, poor cognitive aging, stroke and neurodegenerative diseases of adulthood may have early life origins (McGurn et al., 2008; Shenkin et al., 2009; Hill et al., 2010; Wardlaw et al., 2011; Stoner et al., 2014). Improved understanding of cerebral structural changes across the life course may be useful for studying early life determinants and atypical trajectories that underlie these common problems.

Quantitative volumes from brain structural magnetic resonance imaging (MRI) acquired at different stages of life offer the possibility of new insight into cerebral phenotypes of disease, biomarkers for evaluating treatment protocols, and improved clinical decision-making and diagnosis. The literature presents a clear distinction between methods developed for different ages partly because the computational task is determined by properties of the acquired data and these are age-dependent (Cabezas et al., 2011; Despotovic et al., 2015; Išgum et al., 2015). For example, the infant brain presents challenges to automated segmentation algorithms developed for adult brain due to: wide variations in head size and shape in early life, rapid changes in tissue contrast associated with myelination, decreases in brain water, changes in tissue density, and relatively low contrast to noise ratio between gray matter (GM) and white matter (WM). Therefore, automated segmentation tools for modeling structure over years are limited, and this hampers research that would benefit from robust assessment of the newborn to the adult trajectory.

With regard to methodology, approaches for automatic segmentation of brain MRI can be classified into unsupervised (Cai et al., 2007; Leroy et al., 2011; Weglinski and Fabijanska, 2011; Gui et al., 2012) or supervised (Van Leemput et al., 2001; Fischl et al., 2002; Ashburner and Friston, 2005; Prastawa et al., 2005; Song et al., 2007; Altaye et al., 2008; Weisenfeld and Warfield, 2009; Shi et al., 2010; Kuklisova-Murgasova et al., 2011; Makropoulos et al., 2012; Serag et al., 2012b; Cardoso et al., 2013; Cherel et al., 2015; Moeskops et al., 2015; Wang et al., 2015; Loh et al., 2016) approaches. Supervised approaches have proven to be very successful in medical image segmentation (Aljabar et al., 2009; Lötjönen et al., 2010; Coupé et al., 2011; Rousseau et al., 2011; Kaba et al., 2014). However, as they rely on labeled training data (or atlases) to infer the labels of a test scan, most existing supervised approaches require a large number of training datasets to provide a reasonable level of accuracy and they usually carry a high computation cost due to their requirement of non-linear registrations between labeled data and the test scan (Iglesias and Sabuncu, 2015).

**Figure 1:**
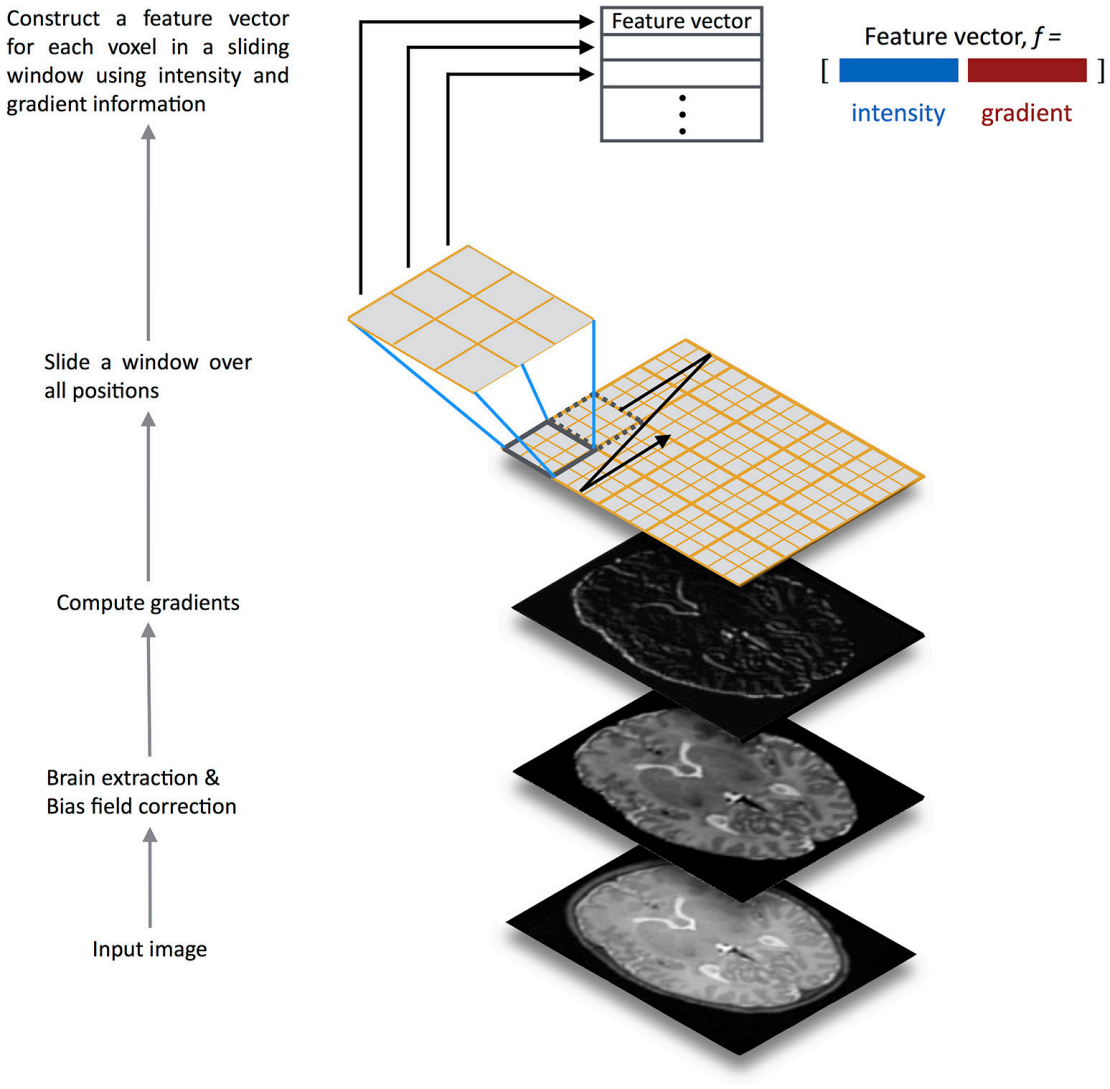
**Overview of the SEGMA feature extraction framework**. The input test image is preprocessed for brain extraction and bias field correction, before computing gradients. Then, a sliding window is scanned across the input image at all positions where a feature vector for each voxel over the window is constructed using intensity and gradient information. The feature vectors are fed into a random forest classifier trained for structure / tissue classification.

To address these challenges, here we describe a method for automatic brain segmentation of MR images, called **SEGMA** (**SEGM**entation **A**pproach). **SEGMA** differs from current supervised approaches in the following ways. First, SEGMA uses a sparsity-based technique for training data selection by selecting training data samples that are “uniformly” distributed in the low-dimensional data space, and hence eliminates the need for target-specific training data (Serag et al., 2016). Second, SEGMA uses linear registration to provide an accurate segmentation (mainly to ensure the same orientation and size for all subjects). This is useful because it reduces computation time compared with most supervised methods which require non-linear registrations between the training images and the target image. Finally, SEGMA uses a machine learning classification based on random forests (Breiman, 2001) where a class label for a given test voxel is determined based on its high-dimensional feature representation. In addition to incorporating more information into the feature set (compared with methods that use voxel intensity information only), we use a sliding window technique that moves over all positions in the test image and classifies all voxels inside the window at once, instead of assigning labels on a voxel by voxel basis. This technique has the advantage of speeding-up the classification process while minimizing misclassifications compared with methods that use a global classifier (Iglesias et al., 2011; Vovk et al., 2011; Zikic et al., 2014). The feature extraction framework is illustrated in Figure 1.

## Materials and methods

### Data and image acquisition

The study includes brain imaging data from 179 subjects, spanning the ages of 0–71 years, from three MRI datasets.

#### Dataset I

The first dataset contained MR images from 66 infants: 56 preterms (mean post-menstrual age [PMA] at birth 29.23 weeks, range 23.28–34.84 weeks) were acquired at term equivalent age (mean PMA 39.84 weeks, range 38.00–42.71 weeks), and 10 healthy infants born at full term (>37 weeks' PMA). None of the infants had focal parenchymal cystic lesions. Participants of the newborns dataset were recruited to a larger study using MRI to study the effect of preterm birth on brain growth and long-term outcome. Ethical approval was granted by the National Research Ethics Service (South East Scotland Research Ethics Committee) and NHS Research and Development, and informed written parental consent was obtained.

A Siemens Magnetom Verio 3T MRI clinical scanner (Siemens Healthcare GmbH, Erlangen, Germany) and 12-channel phased-array head coil were used to acquire: [1] T1-weighted (T1w) 3D MPRAGE: TR = 1650 ms, TE = 2.43 ms, inversion time = 160 ms, flip angle = 9 degrees, acquisition plane = sagittal, voxel size = 1 × 1 × 1 mm^3^, FOV = 256 mm, acquired matrix = 256 × 256, acceleration factor (iPAT) = 2; [2] T2-weighted (T2w) SPACE STIR: TR = 3800 ms, TE = 194 ms, flip angle = 120 degrees, acquisition plane = sagittal, voxel size = 0.9 × 0.9 × 0.9 mm^3^, FOV = 220 mm, acquired matrix = 256 × 218. The image data used in this manuscript are available from the BRAINS repository (Job et al., 2017) (http://www.brainsimagebank.ac.uk).

Reference tissue segmentations for the dataset were generated using an Expectation-Maximization algorithm with tissue priors provided by the atlas from (Serag et al., 2012a,c). Ground truth accuracy of reference neonatal segmentations was evaluated by a radiologist experienced in neonatal brain MRI, who concluded that they were all plausible representations of anatomical classes. Quantitative evaluation of the reference segmentations was performed against manual segmentations from 9 subjects chosen at random. For each subject, three slices (those numbered as 25th percentile, median and 75th percentile of the slices containing brain tissue) were segmented. In order to remove bias toward any particular anatomical plane, three subjects were segmented in the axial plane, three in the coronal plane, and three in the sagittal plane. The quantitative analyses indicated high agreement for all tissues (mean Dice coefficient of 92%).

#### Dataset II

The second dataset contained T1w MRI scans and corresponding manual expert segmentation of 32 structures from 103 subjects (mean age 11.24 years, range 4.20–16.90 years) publicly available from the Child and Adolescent NeuroDevelopment Initiative (CANDI) at University of Massachusetts Medical School (Frazier et al., 2008; Kennedy et al., 2012) (http://www.nitrc.org/projects/candi_share). The data originates from four diagnostic groups: healthy controls (*N* = 29), schizophrenia spectrum (*N* = 20), Bipolar Disorder (*N* = 35), and Bipolar Disorder with psychosis (*N* = 19). The T1w images were acquired using a 1.5T Signa scanner (GE Medical Systems, Milwaukee, USA) with the following parameters: a three-dimensional inversion recovery-prepared spoiled gradient recalled echo coronal series, number of slices = 124, prep = 300 ms, TE = 1 min, flip angle = 25 degrees, FOV = 240 mm^2^, slice thickness = 1.5 mm, acquisition matrix = 256 × 192, number of excitations = 2.

#### Dataset III

The third dataset contained brain images and the corresponding manual expert segmentation of the whole brain into 32 structures from 18 healthy subjects including both adults and children; for the current study, we used only the adult data (*N* = 10, mean age 38, range 35–71 years). The dataset is publicly available from the Internet Brain Segmentation Repository (www.nitrc.org/projects/ibsr) as IBSR v2.0 (Rohlfing, 2012). The T1w images were acquired using the following parameters: scanner/scan parameters unspecified, acquisition plane = sagittal, number of slices = 128, FOV = 256 × 256 mm, voxel size = 0.8–1.0 × 0.8–1.0 × 1.5 mm^3^.

### Preprocessing

For brain extraction, we used the brain masks which are provided with each dataset; except dataset I which was brain extracted using ALFA (Serag et al., 2016). All images from all datasets were corrected for intensity inhomogeneity using the N4 method (Tustison et al., 2010).

### Training data

The number of training examples often must be limited due to the costs associated with procuring, preparing and storing the training examples, and the computational costs associated with learning from them (Weiss and Provost, 2003). Therefore, we use in this work a sparsity-based technique to select a number of representative atlas images that capture population variability by determining a subset of *n*-dimensional samples that are “uniformly” distributed in the low-dimensional data space (Serag et al., 2016). The technique works by first linearly registering (12 degrees of freedom) all images from each dataset to an appropriate common coordinate space, and image intensities are normalized using the method described by (Nyul and Udupa, 2000). For dataset I, the 40 weeks PMA template from the 4D atlas (Serag et al., 2012a) was used as the common space, which is the closest age-matched template to the mean age of the cohort, while datasets II and III were aligned to the common space defined by the International Consortium for Brain Mapping (ICBM) atlas (Mazziotta et al., 2001). Then, all *N* aligned images are considered as candidates for the subset of selected atlases. The closest image to the mean of the dataset is included as the first subset image. The consecutive images are selected sequentially, based on the distances to the images already assigned to the subset. Further details can be found in (Serag et al., 2016).

### Features

We use machine learning to assign a label to all voxels in the test image, based on training a local classifier. Most existing methods for tissue classification only utilize information from voxel intensity, without considering other information. Here, in addition to voxel intensities, we incorporated various gradient-based features. Typically for each voxel *v*, a ten-dimensional feature vector **f**_*v*_ is extracted:

(1) $$f_{v}={\left[\begin{array}{ccc} \begin{array}{cccc} \begin{array}{ccc} \begin{array}{cc} I & I_{x} \end{array} & I_{y} & I_{z} \end{array} & r & \theta & \phi \end{array} & I_{xx} & \begin{array}{cc} I_{yy} & I_{zz} \end{array} \end{array}\right]}^{T}$$

where *I* is the gray scale intensity value, *I*_*x*_, *I*_*y*_ and *I*_*z*_ are the norms of the first order derivatives, and *I*_*xx*_, *I*_*yy*_ and *I*_*zz*_ are the norms of the second order derivatives. The image derivatives are calculated through the filters [−1 0 1]^*T*^ and [−1 2 −1]^*T*^. The gradient magnitude (*r*), azimuth angle (θ) and zenith angle (ϕ) are defined as follows:

(2) $$\begin{array}{r} r=\sqrt{I_{x}^{2}+I_{y}^{2}+I_{z}^{2}} \end{array}$$

(3) $$\begin{array}{r} \theta =\tan^{-1}\left(\frac{I_{y}}{I_{x}}\right) \end{array}$$

(4) $$\begin{array}{r} \phi =\cos^{-1}\left(\frac{I_{z}}{r}\right) \end{array}$$

where *r* ∈ [0, ∞), θ ∈ [0, 2π), and ϕ ∈ [0, π].

### Random forests

In the last decade, random forests (RF) (Breiman, 2001) became a popular ensemble learning algorithm, as they achieve state-of-the-art performance in numerous medical applications (Yi et al., 2009; Huang et al., 2010; Geremia et al., 2011; Mitra et al., 2014; Zikic et al., 2014; Tustison et al., 2015; Pereira et al., 2016). A RF ensemble classifier consists of multiple decision trees. In order to grow these ensembles, often random vectors are generated that govern the growth of each tree in the ensemble. Typically, each tree is trained by combining “bagging” (Breiman, 1996) (where a random selection is made from the examples in the training set) and random selection of a subset of features (Ho, 1998), which construct a collection of decision trees exhibiting controlled variation.

A test sample is pushed down to every decision tree of the random forest. When the sample ends up in one leaf node, the label of the training sample of that node it is assigned to the test sample as tree decision. Then, the final predicted class for a test sample is obtained by combining, in a voting procedure, the predictions of all individual trees. More details on decision forests for computer vision and medical image analysis can be found in Criminisi and Shotton (2013).

### Sliding-window based classification

A sliding window is used to move over all possible positions in the test image, and for each window, the voxels inside the window are classified into different tissues or structures. The vector in equation (1) represents the test sample for one voxel in a window, where the number of test samples is equal to the window size *w*. The training samples come from the voxels of the aligned atlas images that are located at the same location as the voxels belonging to the test window. This means that the number of training samples per window is equal to *k*×*w*, where *k* is the number of training atlases and *w* is the window size, e.g., 5 × 5 × 5, or 7 × 7 × 7, etc.

**Figure 2:**
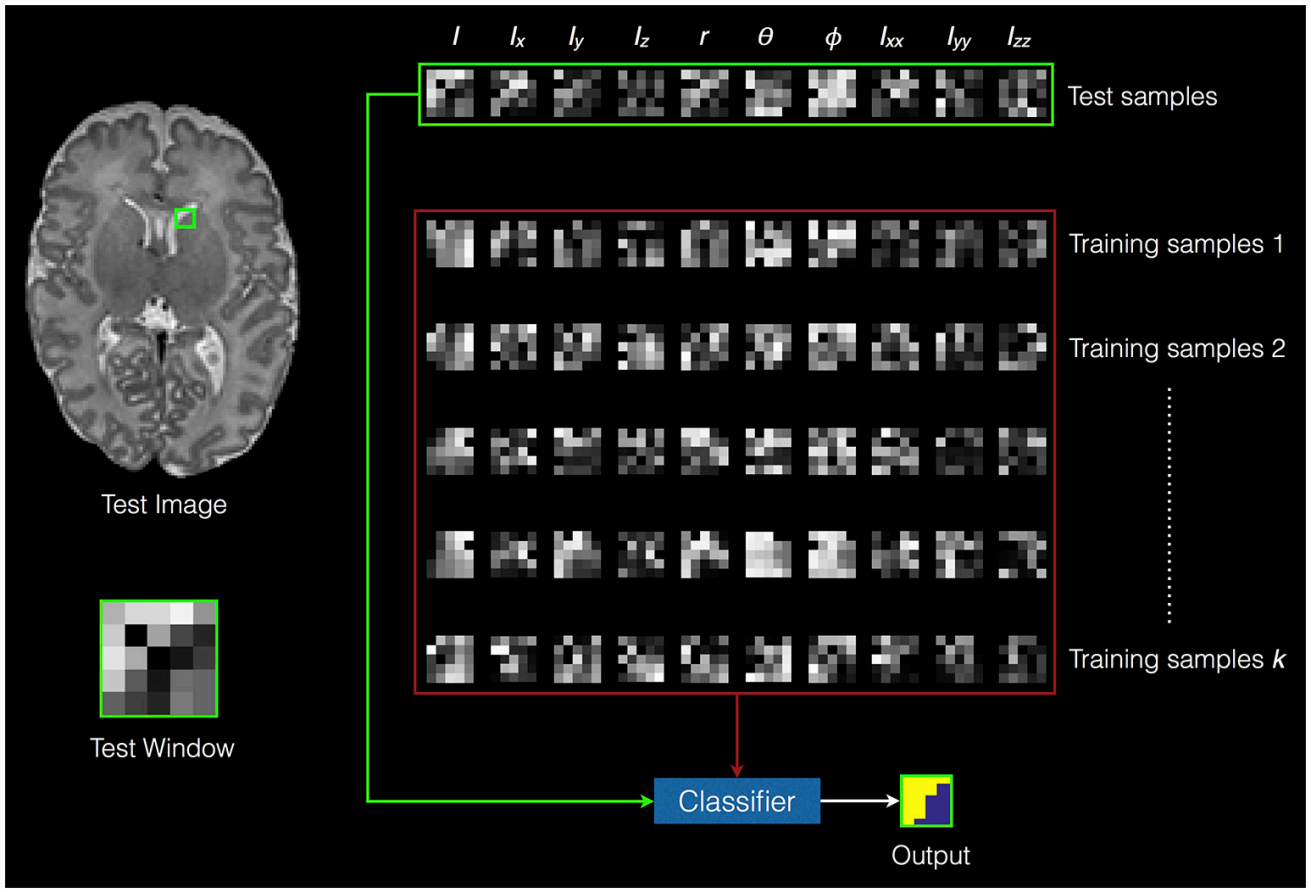
**An example of classifying one test window**. The green square in the test image represents the test window. The green rectangle represents the extracted features from the test window (i.e., test samples). The red rectangle represents the extracted features from training data (i.e., training samples). The voxels inside the test window are classified into different classes based on training the random forest classifier using the training samples.

A local RF classifier is then used to assign each voxel in the test image to a segmentation class. Figure 2 shows an example of classifying one test window. The SEGMA algorithm is summarized in **Algorithm 1**.

**Algorithm 1**. SEGMA algorithm

Set **f**_*v*_ to represent a feature vector for a voxel *v*
Set *c*_*v*_ to represent a segmentation class for a voxel *v*
Set *k* to represent the number of training data
Set *w* to represent the sliding window size
**for** each window *W* **do**
  Construct the training data matrix $T_{W}^{Train}=\{f_{v}^{j}|j\;=\;1,\ldots ,k;\;v\;=\;1,\ldots ,w\}$
  Train the *RF*_*W*_ classifier for window *W* using $T_{W}^{Train}$
  Construct the test data matrix $T_{W}^{Test}=\;\{f_{v}|v\;=\;1,\ldots ,w\}$
  Determine the labels *c*_*v*_ for all voxels inside the test window *W* by applying *RF*_*W*_ to $T_{W}^{Test}$
**end**

### Evaluation

A leave-one-out cross-validation procedure was performed for every dataset. Each subject from a dataset in turn was left out as a test sample and the remaining subjects were used as the training data where a subset of *k* atlases is selected. The comparison between automatic (*A*) and reference (*M*) segmentations was performed using the Dice coefficient (*DC*) (Dice, 1945) which measures the extent of spatial overlap between two binary images, with range 0 (no overlap) to 1 (perfect agreement). The Dice values are expressed as a percentage and obtained using the following equation:

(5) $$DC(A,M)=\frac{2|A\cap M|}{\left|A|+|M\right|}\;\times \;100$$

### Comparison against other methods

We compared SEGMA against commonly used segmentation methods: Majority Vote (MV) (Rohlfing et al., 2004; Heckemann et al., 2006), Simultaneous Truth And Performance Level Estimation (STAPLE) (Warfield et al., 2004). The registration scheme for these methods is based on non-linear image deformation (Rueckert et al., 1999; Modat et al., 2010).

To compare SEGMA against other RF segmentation methods, we implemented a global RF classifier, similar to (Iglesias et al., 2011; Zikic et al., 2014), and experimented training it using intensity and gradient-based features, and intensity feature only. Non-linear registration was used as above to map the training images to the test image coordinate space, and the RF classifier was trained using 100,000 randomly sampled voxels from each training image.

### Statistical analyses

To test for differences between segmentation results, *t*-tests were used for normally distributed data, and Mann Whitney U was used to compare non-normal distributions (Shapiro-Wilk normality test was used). *P* < 0.05 were considered significant after controlling for Type I error using false discovery rate (FDR).

## Results

**Figure 3:**
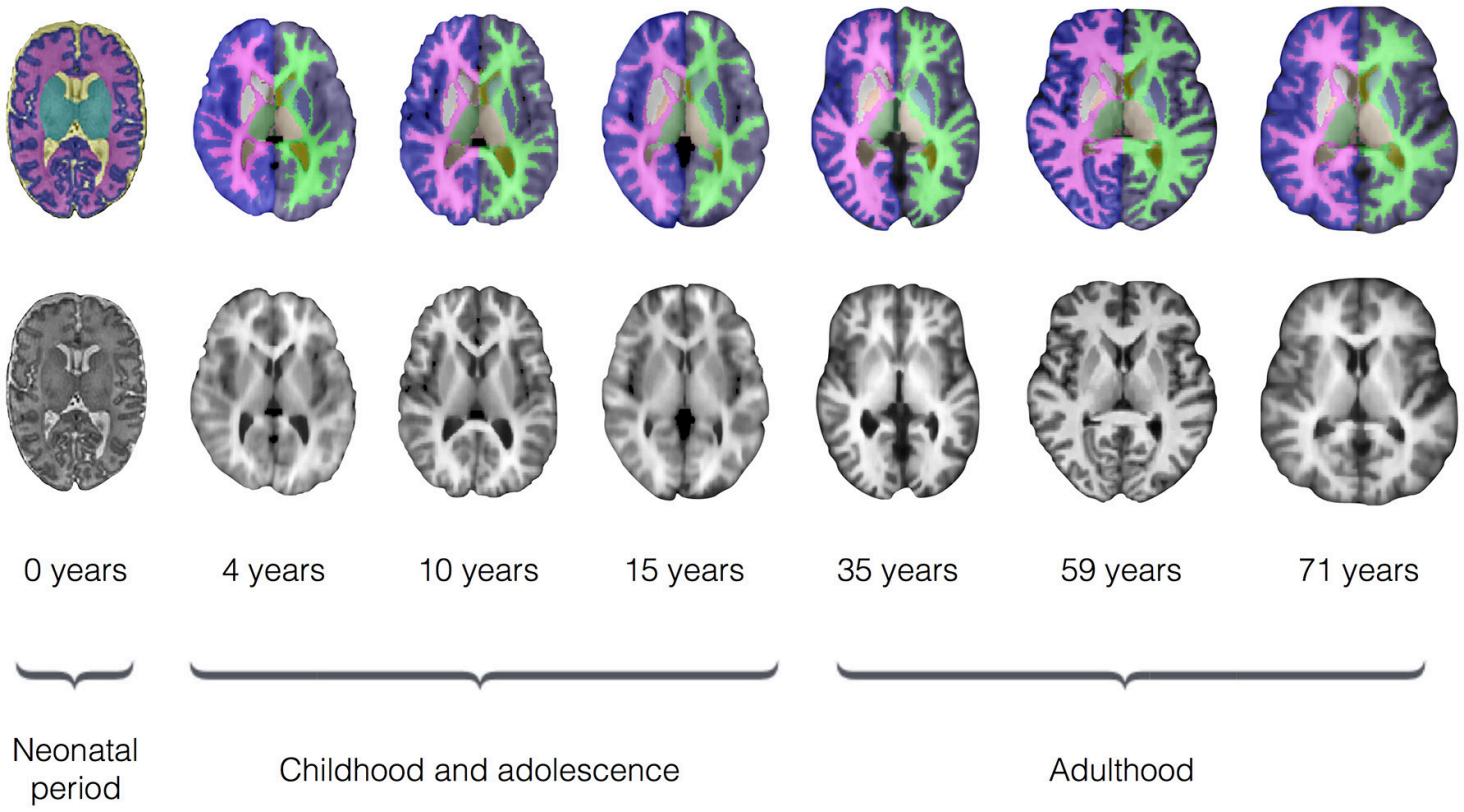
**Examples of brain segmentation results across the life course (axial view) using SEGMA**. The automated segmentation is based on T2-weighted scans for the neonatal period and T1-weighted scans for the rest of growth stages. The images are taken from single subjects at the shown ages, where neonatal period images come from dataset I; childhood and adolescence images come from dataset II; and adulthood images come from dataset III.

**Figure 4:**
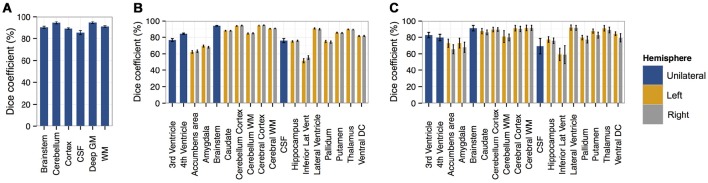
**Bar plots of the Dice coefficient (with standard deviation as error bar) comparing segmentations derived from SEGMA with reference segmentations using (A)** dataset I [neonatal period], **(B)** dataset II [childhood and adolescence], and **(C)** dataset III [adulthood].

To evaluate segmentation performance across the life course, SEGMA was applied to three publicly available datasets that provide MR brain images at different stages of the life course: neonatal period (38–42 weeks gestational age), childhood and adolescence (4–17 years), and adulthood (35–71 years). Figure 3 shows examples of brain segmentation results across the life course, and Figure 4 shows the resulting Dice coefficient (i.e., the agreement between the automatic and reference segmentations).

### Brain segmentation in neonatal period

We first applied the proposed segmentation method to a neonatal cohort (dataset I) consisting of 66 MR images and associated segmentation of the following tissues / structures: brainstem, cerebellum, cortex or GM, cerebrospinal fluid (CSF), deep GM and WM. Quantitative analyses (Figure 4) indicated high accuracy for all tissues and structures with a mean Dice coefficient of 91%.

The highest accuracies obtained for brainstem, cerebellum, deep GM, and WM with mean Dice coefficient of 90–94%, while cortex and CSF had average Dice coefficients of 89 and 85%, respectively.

### Brain segmentation in childhood and adolescence

To examine the performance of SEGMA in childhood and adolescence, we used 103 MR images from subjects aged 4–17 years (dataset II) with associated anatomical segmentation of 32 structures. Quantitative analyses (Figure 4) indicated high accuracy for all tissues and structures with a mean Dice coefficient of 86%. Nine structures had an average Dice coefficient higher than 90%, 7 structures had an average Dice coefficient of 79–89%, and 2 structures had an average Dice coefficient of 51–67%.

### Brains segmentation in adulthood

A dataset (dataset III) consisting of MR images and corresponding anatomical segmentation of 32 structures from 10 subjects (aged 38–71 years) was used to examine the performance of the segmentation algorithm in adulthood. Quantitative analyses (Figure 4) indicated high accuracy of 83%. Seven structures had an average Dice coefficient higher than 90%, 9 structures had an average Dice coefficient of 75–89%, and 2 structures had an average Dice coefficient of 49–57%.

### Comparison against other methods

SEGMA was compared with two commonly used segmentation methods [Majority Vote (MV) (Rohlfing et al., 2004; Heckemann et al., 2006), Simultaneous Truth And Performance Level Estimation (STAPLE) (Warfield et al., 2004)], and other RF-based segmentation methods. SEGMA improved overall segmentation accuracy compared with MV, STAPLE, global-RF-1 (trained using intensity and gradient features), and global- RF- 2 (trained using intensity feature only); Table 1 shows Dice coefficients averaged over all structures, generated by each segmentation method and applied to datasets I, II and III. (*P* < 0.001; after FDR correction).

**Table 1:** **Dice coefficients averaged over all structures for datasets I, II, and III**.

**Dataset**	**SEGMA %**	**Global-RF-1 %**	**Global-RF-2 %**	**MV %**	**STAPLE %**
I	**90.68**	85.29	84.22	86.97	87.01
II	**86.05**	78.98	74.90	81.75	79.17
III	**82.56**	78.75	76.02	77.13	77.54

*SEGMA is compared with MV, STAPLE, global-RF-1, and global-RF-2*.

### Reproducibility

**Figure 5:**
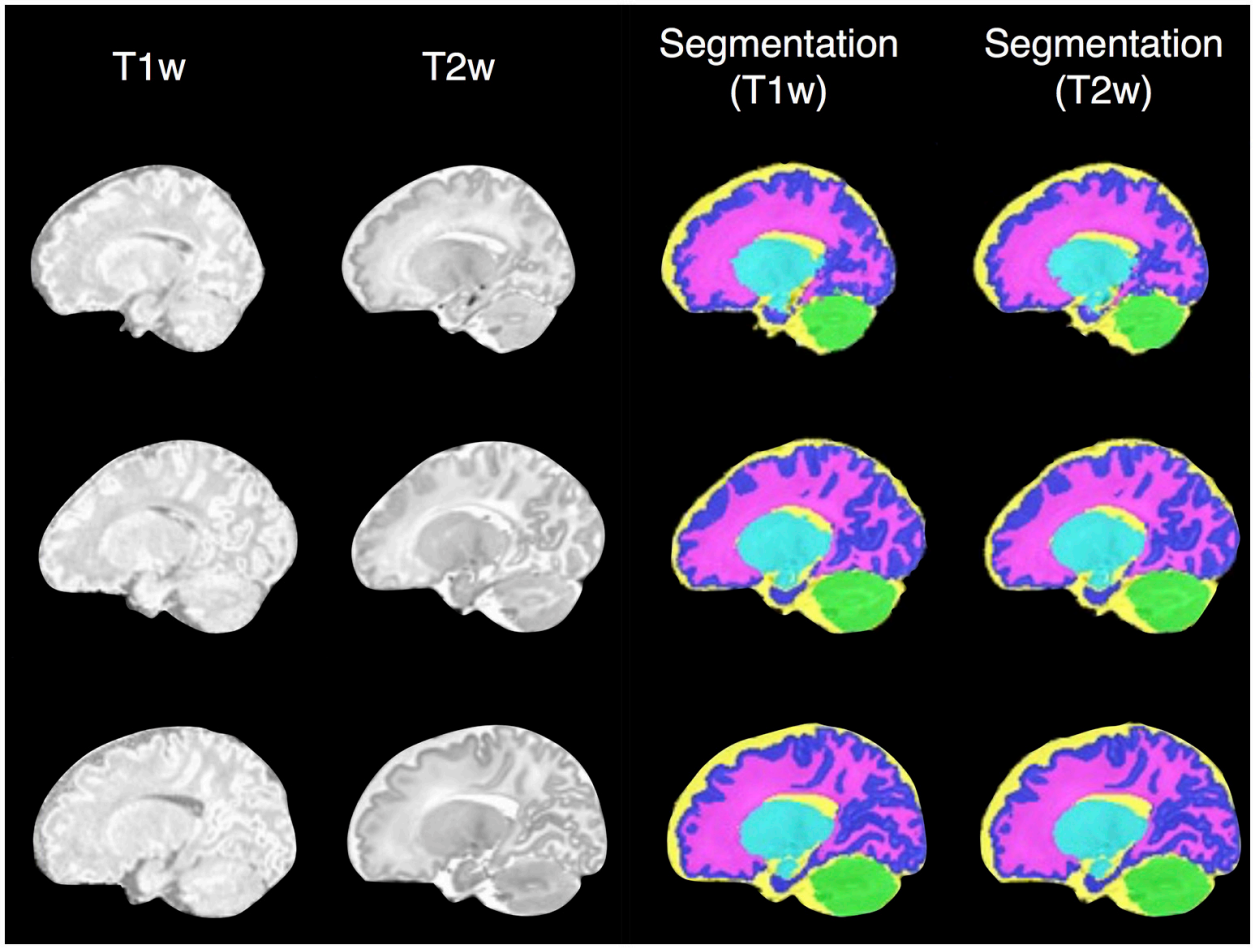
**Examples of SEGMA's output segmentation results (sagittal view) using T1-weighted (T1w) and T2-weighted (T2w) MR individually**.

As dataset I (neonatal period) included T1-weighted (T1w) and T2-weighetd (T2w) MR imaging, we used it to test the reproducibility of SEGMA across different MR modalities by segmenting the newborn brain using information from T1w and T2w data separately (Figure 5). SEGMA provided consistent segmentation results across different structural MRI modalities of the newborn brain. There was no statistically significant difference between mean Dice scores estimated from the two groups (*P* = 0.8977).

### Influence of parameters

We evaluated the influence of size of training data on segmentation accuracy, and found that increasing the size of the training data improves segmentation accuracy, evidenced by the increase in average Dice coefficient from 88% (7% training data) to 91% (30% training data) for neonates, and from 83% (5% training data) to 86% (20% training data) for children and adolescents. From our experiments, 5–10 training images were sufficient to yield accurate results.

Forest parameters such as tree depth and number of samples per leaf node were set according to pervious work (Geremia et al., 2011; Zikic et al., 2014; Wang et al., 2015), and in this work, we only evaluated the influence of number of trees on segmentation accuracy. The number of trees in the forest characterizes the generalization power. As the number of trees becomes large, segmentation accuracy increases, but training time increases and a threshold value is reached after which further improvement is not achieved. In this work, number of trees was set to 10.

With regard to window size, the smaller the window, the longer the classification time. Hence, window size needs to be chosen carefully as it provides a balance between accuracy and speed. Therefore, in this paper, we select the window size as 5 × 5 × 5.

### Relative importance of features

**Figure 6:**
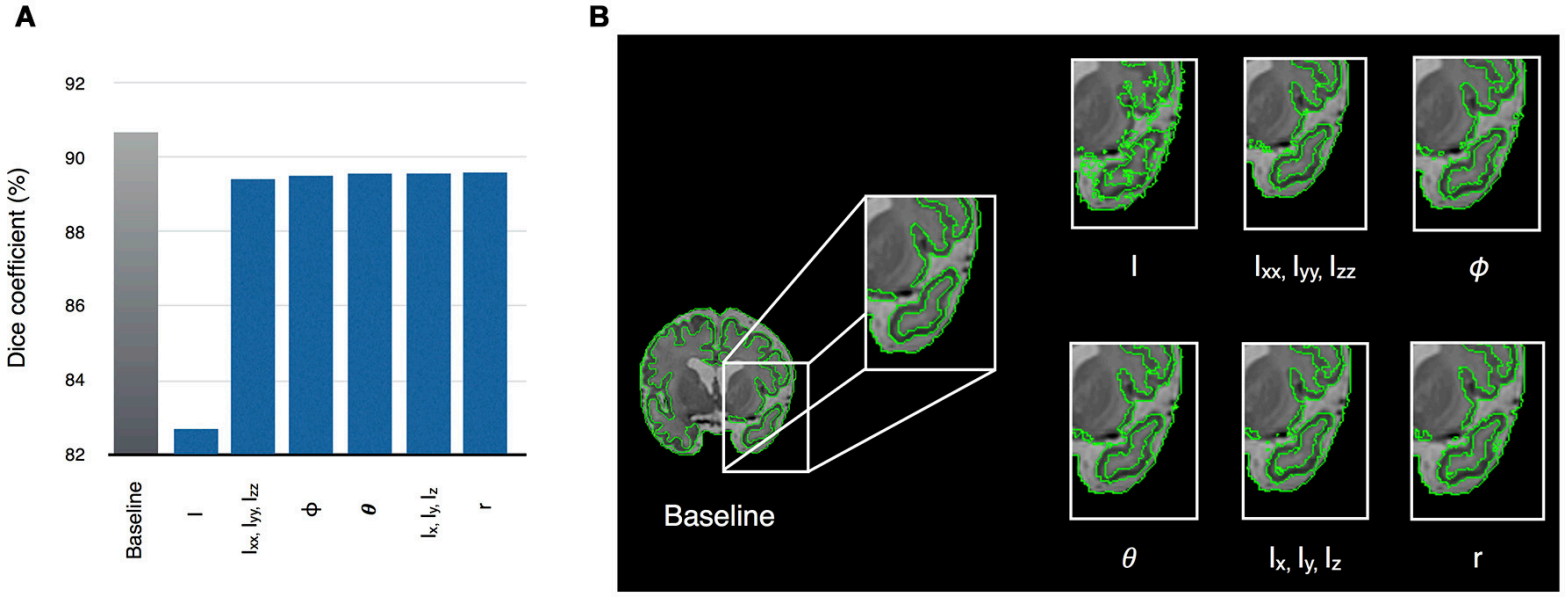
**(A)** Relative importance of each of the ten features, expressed as the segmentation accuracy, on removing the feature from the feature vector. The leftmost bar shows a baseline value—Dice coefficient, when all features are used. **(B)** An example of the automatic segmentation of cortical GM (coronal view), which shows how the dropping of each of the ten features affects the segmentation accuracy. The baseline segmentation is obtained by using all features.

As partial volume effects in neonatal brain MRI present challenges for automatic segmentation methods, we evaluated the influence of each of the features on segmentation accuracy of the neonatal brain (dataset I). This was done by dropping one or a group of the ten features and running segmentation with the remaining features (features of the same type were dropped together). Therefore, an approximation of relative importance of each feature was obtained. Our experiments show that dropping the intensity feature significantly hinders the segmentation accuracy (Figure 6A), whilst the accuracy is improved by incorporating gradient-based features. When all of the features are used, SEGMA yielded higher accuracy than each individual category (*P* < 0.001; after FDR correction). Figure 6B also shows an example of the automatic neonatal cortical GM segmentation and how the dropping of each of the ten features affects the segmentation accuracy.

**Figure 7:**
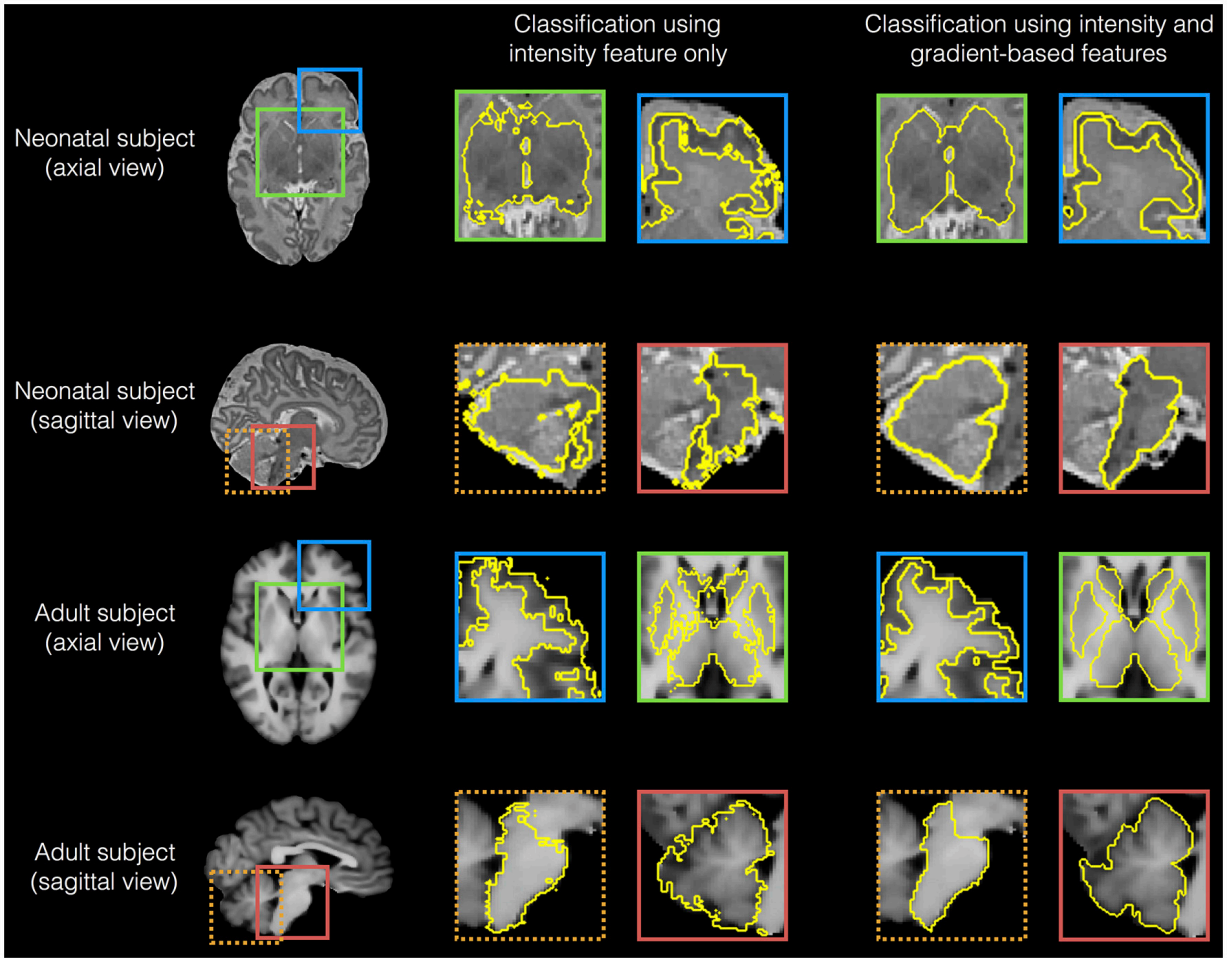
**Examples of edge detection for various regions (cortical gray matter, sub-cortical structures, brainstem and cerebellum) based on using all features (intensity combined with gradients) and intensity gray scale only, for a neonatal (dataset I) and an adult brain (dataset III)**.

We then analyzed the edge detection for various regions based on using all features (intensity combined with gradients) and gray scale intensity only. Figure 7 shows that gradient-based features improved edge detection for various regions of the adult and neonatal brain.

### Computation time

One classification task on a 64-bit iMac® (Intel® Core i7 @ 3.5 GHz × 4.32 GB RAM) takes 5–7 min. The classification has benefited much from the sliding window strategy used. This is because instead of performing the classification in a voxel-wise manner, this is done for a batch of voxels at once. Assuming a window size of 5 × 5 × 5, the classification time is decreased by 125-folds. In addition, multi-core processing or computer clusters could greatly enhance the speed; and then one brain classification could be performed in about (or less than) 1 min.

## Discussion

In this article, we present a new method for MRI brain segmentation (**SEGM**entation **A**pproach, **SEGMA**). SEGMA was evaluated on three different datasets (span the ages 0–71 years) that provide different challenges to the brain segmentation task, and accurate results were obtained at all stages of development.

The method is trained using partially labeled datasets where a relatively small number of manually labeled images from the population under study are sufficient to provide accurate results. It is possible that training the method with a larger dataset might increase the segmentation accuracy. However, our goal was to design a methodology that can provide an acceptable, yet high accuracy result using a small number of training images (and thence a low computation cost).

The relatively lower performance for CSF could be caused by its bordering with GM (which is a complex shape). The boundary between GM and CSF is especially difficult to identify inside the sulci, where it is often poorly visible. In addition, the relatively lower performance for the children and adolescence, and adult datasets compared with the neonatal dataset could be attributable to scanner strength. Yet, the results obtained are comparable with those obtained using other methods tested on the same datasets (Rousseau et al., 2011; Zikic et al., 2014).

SEGMA uses a local RF classifier (trained by information from neighboring voxels in the same window) to assign a label to each voxel, which makes it less susceptible to classification errors such as the partial volume misclassification on the CSF-GM and CSF-background boundaries (Kuklisova-Murgasova et al., 2011; Cardoso et al., 2013; Išgum et al., 2015; Moeskops et al., 2015). We chose to use random forests as the classification technique since they naturally handle multi-class classification problems and are accurate and fast (Huang et al., 2010; Geremia et al., 2011; Criminisi and Shotton, 2013). Also, the sliding window plays an important role in significantly speeding up the classification task (compared to voxel-wise approaches).

The method provides an accurate segmentation using only linear registration, which ensures the same orientation and size for all subjects. This is an advantage compared with most supervised methods, which require non-linear registrations between the training images and the test image which increases segmentation time to several hours thereby compromising clinical utility (Iglesias and Sabuncu, 2015). SEGMA also has the advantage of providing an accurate segmentation using a single modality (which is important as the available data might be limited to one modality), and features that characterize object appearance and shape (intensity and gradients). However, the method is flexible and new features can easily be added to the high-dimensional feature vector.

To conclude, we present a method for segmentation of human brain MRI that is robust and provides accurate and consistent results across different age groups and modalities. As SEGMA can learn from partially labeled datasets, it can be used to segment large-scale datasets efficiently. The idea of SEGMA is generic and could be applied to different populations and imaging modalities across the life course. SEGMA is available to the research community at http://brainsquare.org.

## Author contributions

AS designed and performed the experiments, and wrote the manuscript; AS, JB, and AGW analyzed output data; ET, RP, and SAS recruited patients; GM and SIS acquired imaging data. All authors approved the final submitted version, and agreed to be accountable for its content.

## Funding

This work was supported by the Theirworld (http://www.theirworld.org), NHS Research Scotland, and NHS Lothian Research and Development. This work was undertaken in the MRC Centre for Reproductive Health which is funded by the MRC Centre grant MR/N022556/1.

### Conflict of interest statement

The authors declare that the research was conducted in the absence of any commercial or financial relationships that could be construed as a potential conflict of interest.
